# Acute Cardiac Care for People With Severe Mental Illness Following a Myocardial Infarction Among People With a Severe Mental Illness: A Qualitative Study

**DOI:** 10.1192/bjo.2024.87

**Published:** 2024-08-01

**Authors:** Amanda Vettini, Debbie Cavers, Sandosh Padmanabhan, Daniel Smith, Caroline Jackson

**Affiliations:** 1 University of Edihnburgh, Edinburgh, United Kingdom; 2 University of Glasgow, Glasgow, United Kingdom

## Abstract

**Aims:**

To understand the challenges and barriers experienced by health-care professionals (HCPs) in providing acute cardiac care to patients with severe mental illness (SMI) (schizophrenia, bipolar disorder or severe depression) admitted to hospital following a myocardial infarction (MI).

**Methods:**

Semi-structured 1:1 videocall interviews with 12 HCPs in two central-Scotland Health Boards involved in delivering pre-/hospital acute care for a MI (paramedics, cardiology/A&E nurses, cardiology/A&E doctors). Interviewee recruitment was via clinical and research networks and newsletters e.g. the Scottish Ambulance Service, the Royal College of Nursing and Royal College of Physicians and through professional connections. Interviews were audio-recorded, transcribed verbatim and analysed thematically drawing on Braun & Clarke and using NVivo software.

**Results:**

HCPs identified a number of challenges/barriers to providing optimal post-MI acute cardiac care to patients with a SMI across 3 key themes: patient-related; practitioner-related and system/environment-related. Core patient-related challenges/barriers included: diminished patient history capacities especially relating to chronology; the time-consuming nature of effective HCP-patient communication and engagement; medication and intervention concordance concerns and challenging patient behaviour including physical and verbal aggression or severe distress.

Practitioner-related challenges/barriers were: fears of appropriately managing patient behaviour; stigma towards patients with a SMI (putatively arising from knowledge deficits or generational/age-related effects); staff burnout due to length of service and pressures from extreme workloads.

Systemic issues included insufficient staffing precluding the additional time required for effective communication and the distressing nature of hospital environments for patients with a SMI. Side rooms were not routinely available even though these were identified as improving the environment for some patients. A core systemic finding, cited by all interviewees, was the lack of adequate training provision on caring for patients with a SMI. Additional system-level findings were degrees of challenges accessing input from the hospital psychiatric team especially outwith standard hours and problems obtaining rarer psychiatric medications potentially impacting patients’ mental health stability.

Positive findings included that HCPs are generally enthusiastic about providing high quality care to this patient group and to seek help with this. Some HCPs indicated that caring for mentally stable patients with a SMI does not differ from the general population.

**Conclusion:**

Although HCPs aspired to providing optimal acute cardiac care for this patient group, patient-level, professional and systemic barriers often make this challenging. A key area for improvement is enhancing staff training in caring for patients with SMI, ideally delivered in-person.

